# Feasibility and safety of an algorithm for the management of adhesive small bowel obstruction

**DOI:** 10.1007/s00423-025-03820-7

**Published:** 2025-09-03

**Authors:** Dimitri Chappalley, Nicolas Dupasquier, Gabriel Heierli, Maria Teresa Torres, Dieter Hahnloser, Fabian Grass, Martin Hübner

**Affiliations:** Department of Visceral Surgery, University Hospital Lausanne (CHUV), University of Lausanne (UNIL), Rue du Bugnon 46, Lausanne, 1011 Switzerland

**Keywords:** Small bowel obstruction, Adhesion, Nasogastric tube, Conservative treatment

## Abstract

**Introduction:**

This single-center, prospective cohort study assessed the feasibility and safety of a structured management protocol (Ileus Management Algorithm Protocol: I-MAP) for patients with adhesive small bowel obstruction (ASBO).

**Method:**

Among 63 patients initially admitted for ASBO, 40 met the inclusion criteria and were managed in line with I-MAP, incorporating nasogastric tube (NGT) output and water-soluble contrast (WSC) administration within a standardized decision-making framework.

**Results:**

The study’s primary outcome, protocol adherence, was achieved in 87.5% (35/40) of patients, demonstrating the protocol’s feasibility. 27/35 patients (77%) had ASBO resolution with conservative management. Eight patients required surgery, mostly due to lack of improvement after 72 hours of conservative management. Complications were minimal, with only two cases of bronchial aspiration. No patient died within 30 days of conservative management.

**Conclusion:**

This standardized algorithm using pre-defined NGT output values proved feasible and safe, standardizing care and providing structured guidance in clinical decision-making. Larger trials are necessary to confirm efficacy and explore patient-reported outcomes.

**Supplementary Information:**

The online version contains supplementary material available at 10.1007/s00423-025-03820-7.

## Introduction

Adhesive small bowel obstruction (ASBO) is a leading cause of both emergency admission and surgery, accounting for 51% of emergency laparotomies in the UK [1, 2]. Related costs are important, averaging €40,467 per patient according to a recent Swedish study [3].

Initial management is mostly conservative, achieving non-operative resolution in 70–80% of patients [4, 5]. Guidelines generally recommend 3–5 days of non-operative management depending on clinical presentation and evolution [6]; further delaying surgery increases the risk of bowel resection, morbidity, and mortality [1, 7]. Computed tomography (CT)-scans are highly accurate for diagnosis and localization of obstructive sites [8] but fall short to reliably predict resolution of ASBO treatment under conservative treatment vs. need for surgical treatment. Despite multiple recommendations and evidence of safety regarding the use of water soluble contrast agents (WSC) [1], it is still underutilized [9–11]. Despite the widespread use of nasogastric tubes (NGT) in ASBO management, none of the studies rely on nasogastric output or assess its clinical relevance.

Since conservative management and duration are poorly defined, standardization may help with guidance in clinical practice and improve outcomes.

The aim of the present study was to assess the feasibility and safety of a comprehensive management protocol for ASBO (I-MAP).

## Materials and methods

### Study design

This is a single-center prospective cohort study on patients with ASBO conducted at Lausanne University Hospital (CHUV). The study aimed to assess the feasibility and safety of the ASBO itinerary and to gather preliminary information on its efficacy. Approval was obtained from the Research Ethics Commission on human beings of the Canton de Vaud (CER-VD 2022 − 01420). The study adhered to the ethical principles of the Declaration of Helsinki [12] and the principles and procedures for integrity in scientific research involving human beings. All participants provided general consent. This study adheres to the criteria of the STROBE checklist for cohort studies [13] (Table S1).

### Participants

Eligible were adult (≥ 18y) patients admitted for CT-diagnosed acute ASBO. Exclusion criteria were the need for immediate emergency surgery due to complicated disease presentation at admission (strangulated hernia, malignant obstruction, peritonitis etc.), previous intra-abdominal surgery or conservative management of ASBO within the last 30 days, pregnancy and lack of general consent.

### Intervention

The proposed algorithm was based on a literature review and actual guidelines on the diagnosis and management of acute ASBO (Table 1) with a special emphasis on logistic feasibility, acceptability by staff and patients, and objective and unambiguous criteria [8, 17, 18].

**Table 1:** Comparison between I-Map protocol and other guideline

	Bologna Guidelines 2018 [1]	Current study 1-MAP 2024	Loftus T 2015 [14]	Zielinski MD 2017 [10]	Long S 2019 [15]	Dombert L 2020 [16]	Elsolh B 2021 [11]	Krielen P 2023 [9]
Country	World society of emergency surgery	Switzerland	USA	USA	USA	USA	Canada	Netherlands
Type of studies	guidelines	prospective	retrospective	prospective	retrospective	Retrospective Pre and post-protocol	prospective	prospective
Type of SBO	aSBO	aSBO	aSBO/hernia	aSBO	aSBO	aSBO	aSBO	aSBO
Exclusion criteria	Strangulation, bowel ischemia, Peritonitis	Strangulation, peritonitis, bowel ischemia, Hernia, previous abdominal surgery < 30 days	No abdominal surgery, mesenteric edema, pneumatosis, perforation, closed-loop obstruction, or swirl sign with free air	Strangulation, peritonitis, hernia, abdominal/pelvic malignancy, closed-loop obstruction, previous abdominal surgery < 6 weeks.	Peritonitis, non-adhesive source of partial small bowel obstruction, previous abdominal surgery < 30 days	bowel strangulation, internal or incarcerated hernia, a malignant obstruction	Free fluid, ischemia, closed loop, Malignant disease, IBD, hernias	Neoplasm, ventral hernia, volvulus, stenosis, internal herniation
NGT Decompression time		NGT in suction, decompression > 6hrs	NGT decompression > 6hours,		NGT in suction	NGT decompression,	NGT in suction, decompression l hour,	
X-ray assessment	WSC and X-ray after section 24–36hours	WSC, no X-ray mandatory	WCS and X-ray after 4, 8, 12, 24 hours	WSC and X-ray after 8hours	WSC and X-ray after 4–24hours	WSC and X-ray after 24hours	WSC and X-ray after 4 and 24hours	
Maximal length of non.operative protocol	72hours	72hours	< 2 days	4–5 days	< 2 days	72hours	< 2 days	8
Other decisional criteria	No WSC in Colon after 24–36hours → Surgery	Based on Symptoms, no X-ray mandatory, > 72hours → Surgery	No WSC in colon > 24hours → Surgery	No WSC in colon > 8hour → Surgery	No WSC in colon > 24hour → Surgery	No WSC in colon > 24hour → Surgery	No WSC in colon > 24hours → consider surgery	
N’ of patients		40	102	316	1302 (103post/1199pre)	767 (296 post/471 pre)	48	311
Use of WSC agent	Yes	Yes	Yes	55% Yes, 45% No	8% Yes, 92% No	61% Yes (post-protocol), 39% No (pre-protocol)	Yes	26% Yes, 74% No
Evaluation of protocol follow-up based on NGT volume output	No	Yes	No	No	No	No	No	No
Median los	-	6 days		4 days (WSC) 5 days (WSC)	5 days	4 days(post-protocol) 7 days(pre-protocol)	3 days	8 days
No surgery		5 days	3 days			3 days(post-protocol) 5 days(pre-protocol)		3 days
Surgery		13 days	11 days			9 days(post-protocol) 16 days (pre-protocol)		11 days
NGT reinsertion	-	4	-	-	-	-	-	-
Rehospitalisation for ileus	-	15%	11%	-	-	-	29%	15%
Compliance		87.5%	99%	-	-	-	71%	-

The algorithm was presented to the entire surgical team during different staff meetings with a final decision of approval made in March 2023. The nursing team also received a dedicated presentation in Mai 2023 before the study launch. Additionally, pocket cards were printed and laminated for all junior doctors.

A nasogastric tube (NGT) was inserted in all patients in the emergency department with concomitant fluid resuscitation. In accordance with the Bologna Guidelines [1], all patients underwent computed tomography (CT) imaging in order to avoid any complicated ASBO. Laboratory tests (leukocytosis exceeded 10 G/L and C-reactive protein (CRP) levels greater than 75 mg/L), lactate or electrolytes disturbances have low evidence and were not relevant for decision making [14]. The primary objective of the CT scan was to exclude unrelated pathologies and assess radiological signs indicative of bowel ischemia or other forms of intestinal compromise. In case of complicated ASBO, patients underwent immediate surgery. After transfer to the surgical ward, NGT (Salem sump Nasogastric tube Size 12-18 fg) was connected to active continuous suction at – 20mmHg (3 kPa) in line with our institutional practice. Decompression with the NGT had to have taken place for a minimum of 12 h before starting the algorithm.

**Fig. 1:**
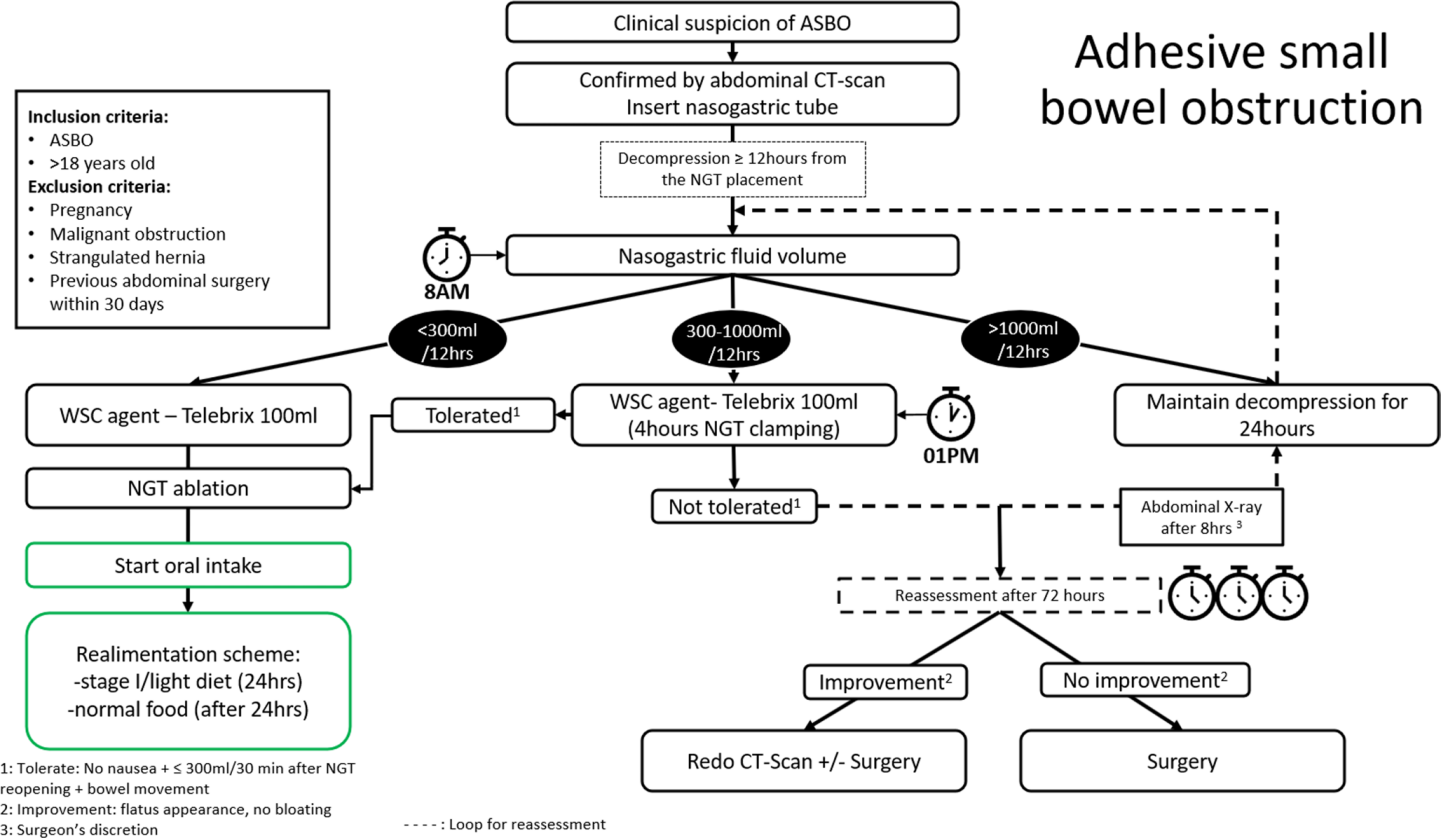
I-MAP, Ileus Management Protocol used for adhesive small bowel obstruction. SBO: small bowel obstruction, NGT: nasogastric tube, WSC: water soluble contrast

Further management followed the comprehensive algorithm established by the core team (Fig. 1). Management decisions were mainly based on NGT output over the last 12 h assessed at 7 a.m., coinciding with the shift change of the nursing team. During morning rounds, patient management was defined according to NGT output: group 1: < 300 ml, group 2: 300–1000 ml, and group 3: >1000 ml.

For group 1 and 2, a WSC medium (TELEBRIX gastro sol ^®^ (Guerbet AG, Zürich)), containing ioxitalamique acid 300 mg/ml) was administered at a single dose of 100 ml in an attempt to foster resolution of clinical symptoms. In group 1, the NGT was removed after WSC administration. In contrast, group 2 kept the NGT in place for 4 h until ASBO resolution, good tolerance of the contrast agent, or plain abdominal x-ray confirmation [11, 19–22]. In Group 2, the surgeon decided whether an abdominal X-ray was mandatory. If experiencing nausea, totaling > 300 mL/30 min output after NGT reopening, or experiencing a lack of bowel movement, the patient was reevaluated the next morning.

For group 3, if totaling > 1000 ml/24hrs NGT output, decompression was maintained an additional 24 h. After an observation period of 48 h, the patient was prepared regarding potential surgical management.

Patient condition was reassessed 4 times daily by nursing staff and twice by medical staff (morning and afternoon rounds). In case of clinical and biological deterioration indicating a risk of small bowel distress, the patient was immediately taken to the operating room. This was neither considered a failure of conservative treatment nor a failure of the protocol. Patients requiring surgical intervention were scheduled for surgery; the degree of urgency was left to the surgeon’s assessment within an intended intervention delay of 12 h at most. Patients with NGTs in place beyond 72 h [6, 23] were considered as refractory ASBO patients and hence scheduled for surgery. Only patients with evident clinical improvement despite NGT output > 300 ml underwent a 2nd CT-scan evaluation. Otherwise, surgical exploration was proposed.

### Outcomes/study endpoints

The primary outcome was the feasibility of the I-MAP algorithm, measured by the ratio of patients treated according to the algorithm to the total number of eligible ASBO patients during the study period. A protocol adherence rate of 85% was defined as minimal threshold to demonstrate feasibility [24, 25]. The threshold was inspired by ERAS studies and infectious diseases literature, demonstrating a cut-off of 80% was most beneficial to seek a clinical benefit. Adherence, defined as compliance with all key steps of the algorithm, was assessed for every patient. Any violation—such as an incorrect timing of reassessment, deviations within the decision branch, or failure to remove the NGT when indicated—was considered non-adherence.

Of note, patients requiring surgery were not considered as dropouts if they were managed according to the algorithm. Secondary outcomes included the rate of NGT reinsertion, total duration of NGT decompression, length of stay (LOS), time from surgical indication to surgery, and specific complications including pneumonia, bronchoaspiration, and need for surgical bowel resection. Patients were followed-up until 3 months post discharge.

### Data synthesis and analysis

Categorical variables were expressed as frequencies and percentages, and continuous variables as median (range) and mean (± SD). Data analysis was performed using Microsoft Excel 365 (Microsoft Corporation, Redmond, WA, USA Software Group, Chicago, IL, USA).

## Results

### Participants

**Fig. 2:**
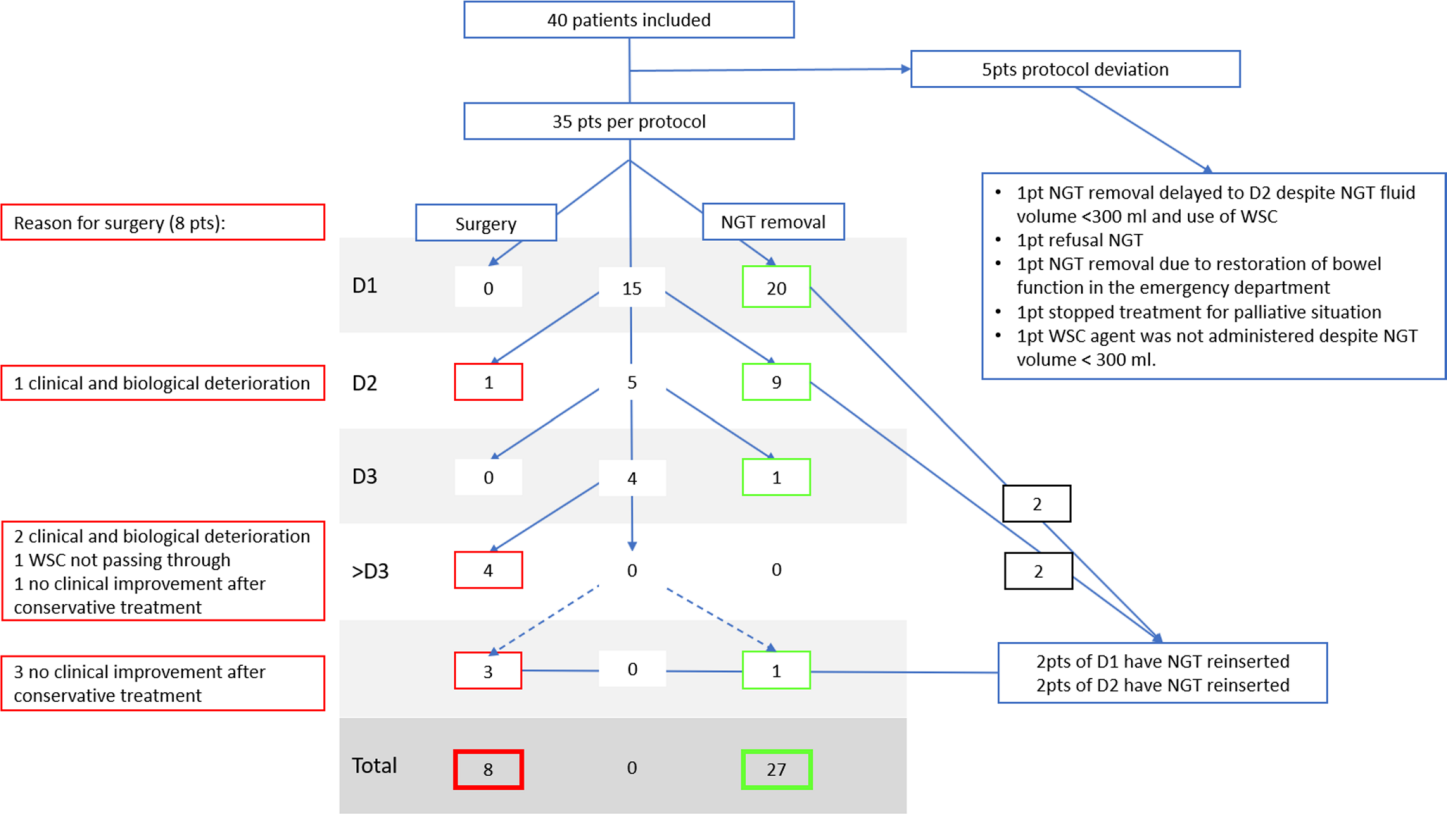
Flowchart diagram illustrating patient recruitment for management according to the ASBO protocol. It also outlines the primary and secondary outcomes. ASBO: adhesive small bowel obstruction. NGT: Nasogastric Tube, WSC: Water soluble contrast, Pt(s): patient(s)

Patient recruitment is summarized in the flowchart diagram (Fig. 2). From June 15, 2023, to March 1, 2024, 63 patients were admitted from the emergency department for acute ASBO. Twelve patients declined general research consent, and five patients needed immediate emergency surgery for strangulation (*n* = 3), peritonitis (*n* = 1), and ischemia (*n* = 1), while 6 patients had another final diagnosis according to abdominal imaging (intraluminal obstructions, carcinosis, ileostomy stenosis). The remaining 40 patients were hospitalized for conservative management and constituted the final study cohort.

### Patient characteristics and operative data

Patient characteristics are summarized in Table 2. The median age was 71 years [30–95], and 48% were female. Sixteen patients presented with recurrent ASBO, of which eight had previous surgery for ASBO. Thirty-eight patients (95%) had previous abdominal surgery, with 29 (73%) having undergone open surgery.

**Table 2:** Baseline characteristics, comorbidities, and previous operative data of the 46 included ASBO episodes

Demographics and prior surgeries	
Age, y, median (range)	71 (30–95)
Female sex, n (%)	19 (48)
BMI kg/m2, median (range)	24.8 (13.2–41.0)
ASA classification, median (range)	3 (2–4)
Previous ASBO, n (%)	16 (40)
Previous surgical treatment of ASBO, n (%)	8 (20)
Number of previous abdominal surgery, median (range)	2 (0–7)
Patients with previous abdominal surgery, n (%)	38 (95)
Open, n (%)	29 (73)
Laparoscopy only, n (%)	9 (22)

### Primary outcomes

Overall, 35 out of 40 patients (87.5%) were treated according to the protocol (Fig. 2).

The causes for protocol non-compliance were refusal of NGT placement (*n* = 1), NGT removal above threshold (*n* = 2), or delayed administration of WSC upon decision of the consultant surgeon in charge (*n* = 2). Out of these 5 patients with non-adherence to the pathway, 4 were successfully managed conservatively, and 1 patient refused further care and died during hospitalization (Fig. 2).

### Secondary outcomes

Overall, 35 patients (87.5%) were treated per protocol (pp). Surgery was needed in 8 patients (23%). The decision to proceed to surgery was made during the first three days in 1 patient because of clinical and biological worsening, while 7 patients were operated after 72 h. The indications for surgery were the lack of improvement after conservative treatment for all 7 patients as planned in the protocol, including clinical and biological deterioration (*n* = 2) and failure of contrast agent passage on repeated CT-scan (*n* = 1). Median delay from indication to surgery was 28 h (4–96 h) due to non-availability of the OR.

Among the eight patients who required surgery, two experienced enterotomies during adhesiolysis, one required reoperation due to a missed enterotomy, and another needed NGT reinsertion for persistent paralytic ileus following initial NGT removal. The postsurgical median LOS was 7 days.

On the contrary, 27 patients (77%) experienced ileus resolution with conservative management. NGTs were removed in most patients after day 1 or 2, as displayed. Re-insertion of NGT occurred in 4 patients, and 3 of them were ultimately operated on. The NGT reinsertion was needed 4 h after NGT removal (*n* = 1), 24 h after NGT removal (*n* = 2), and 48 h after NGT removal (*n* = 1), respectively (Fig. 2). Bronchial aspiration occurred in 1 patient. The patient was diagnosed with aspiration pneumonia three days after the insertion of the NGT, on the day it was removed [16, 26].

Six patients (15%) were readmitted for recurrent ASBO. One patient was readmitted and required immediate surgery 2.5 months after the initial pp treatment. One patient was readmitted 24 days after the previous episode and eventually treated surgically. She again failed to respond to conservative management and subsequently underwent surgery for a new adhesion. Four other patients were managed pp again, 28days, 26 days, 26 days, and 20 days after the initial episode, respectively.

Table 3 summarizes clinical outcomes of the study cohort.

**Table 3:** ASBO Length of stay and NGT characteristics compared with respected and non respected protocols

	All episodes (N=40)	Respected protocol (N=35)	Non respected protocol (N=5)
ASBO resolution, n (%)	31 (78)	27 (77)	5 (100)
Length of stay (LOS)			
LOS, day, median (range)	6 (2 - 53)	7 (2–53)	6 (5 - 14)
LOS conservative, day, median (range)	5 (2 - 37)	5 (2–37)	6 (5- 14)
LOS surgery, day, median (range)	13 (7 - 53)	13 (7–53)	-

## Discussion

The proposed algorithm was successfully implemented in our institution, demonstrating both practicality and safety. This pathway is simple and pragmatic and might hence be used also in other settings and institutions. Clinical benefits as compared with standard case-by-case care need yet to be demonstrated.

Standardization of care is beneficial from a caregiver (decisional framework for guidance, simplification of care) and patient (defined treatment goals and understanding/anticipation of next steps) perspective [1, 23]. In order to improve outcomes, algorithms should rely on best available evidence rather than mere clinical judgement. The present algorithm was defined after thorough review of the evidence-based ASBO literature and recent guidelines [1, 6, 19–21, 27], while also taking into account institutional practice and organizational issues. We specifically tried to assess a gap in the literature regarding timing of NGT ablation, specification of clinical decision making rather than based on radiologic imaging studies and protocol adherence. As a priority, the safety of the suggested management strategy had to be affirmed, and particular importance was attached to acceptability of the algorithm not only for care teams but also for the patient. Even though not specifically assessed in the setting of this pilot project, periodic and systematic reevaluation of treatment goals may contribute to better understanding of treatment decisions [28]. A conservative approach can be justified for up to 72 h, after which the ASBO is unlikely to resolve [1, 6, 23]. The algorithm was validated daily, and the patient was checked upon up to 4 times daily, with measures taken according to predefined NGT output thresholds. In our hospital, it was very difficult to obtain NGT output during the 12 h following NGT insertion, apart during morning rounds. This pragmatic and systematic care strategy is designed to support clinical decision-making and streamline logistics, with medical and nursing staff present during morning rounds to plan next steps. The 300 mL and 1000 mL thresholds are easy to remember, supporting clear and reproducible decision-making. The thresholds were chosen arbitrarily based on institutional experience and practice, as no previous studies have implemented volume cut-offs into their definitions. The clinical criteria for the success of conservative treatment are also clearly defined and appropriately based on the literature [29] bowel movement, passage of flatus, and absence of nausea. Surgical intervention should be considered in case of clinical or biological deterioration, with a 72-hour decision checkpoint to optimize efficacy in both ASBO resolution and patient recovery [1]. The algorithm proposed by the Bologna guidelines is difficult to implement in a busy real-world setting because the emergency department will perform most of the CT-scans without oral contrast agents, while re-assessment of output from insertion at variable timepoints would arguably be challenging [1].

Different algorithms for ASBO management have been proposed before [1, 14, 30]. Similar to our strategy, WSC is integral part of most treatment protocols for both diagnostic (localization of mechanical obstruction) and therapeutic (hyperosmolar laxatif) purposes [30]. Several studies have demonstrated WSC to significantly impact the course of ASBO by aiding in surgical decision-making [20, 21].

Water-soluble contrast agents, such as Gastrografin^®^, have well-documented diagnostic and therapeutic roles in ASBO management [19]. Despite strong evidence and guideline recommendations, their use remains limited in practice without Guidelines [9–11]. Our protocol integrates WSC early in the decision pathway., While Gastrografin^®^ was used in this study, similar agents can be considered interchangeably for clinical use.

As a distinctive feature, our protocol suggests repeated assessment ± WSC administration every morning at fixed hours depending on drainage thresholds until ASBO resolution. Following the Toronto 2019 ASBO guidelines [11], abdominal x-rays could be performed 4 to 8 h after administration. The lack of consistency in the use of abdominal radiography to guide surgical decision-making led us to propose radiography as an option rather than a mandatory step [19].

Table 1 compares different treatment algorithms to our suggested protocol [1, 9–11, 14–16].

The present algorithm demonstrated its feasibility reaching the 85% compliance threshold. This level of compliance was considered relevant based on similar studies on beneficial compliance thresholds [24, 25]. Other studies described similar compliance rates of 77% [11]. From the perspective of symptom resolution, the present study aligns well with the literature reporting need for surgery in up to 30% of patients, while the remaining patients can be successfully managed conservatively. Bronchoaspiration and pneumonia representing potentially severe complications of conservative ASBO treatment were observed in only one patient, supporting the safety of the suggested management strategy.

Short time to ASBO resolution, prompt reevaluation in case of clinical deterioration and avoiding surgery are arguably priorities in ASBO management from the patients’ perspective [28]. From a caregiver standpoint, standardized algorithms help to improve both team efficacy and satisfaction [31]. Finally, shorter length of hospital stay promotes patient turnover and may ultimately help to decrease costs to society [32].The median length of stay of 7 days in this series compares well to the literature [9, 14]. However, the importance of these quality metrics with particular emphasis on PROMs such as short hospital stay, prompt recovery and quality of life need to be further evaluated in future trials.

This study has several limitations related to its sample size which was not powered to assess clinical outcomes. Furthermore, the heterogeneity of ASBO patients highlights challenges related to a “one size fits all approach”. The absence of predefined thresholds for clinical evaluation and the lack of systematically reported values for NGT output represent a limitation of our study, but also provide an initial framework for implementing this type of clinical assessment. Local habits and expectations need to be considered when creating standardized algorithms. It is important to note that due to these local logistics and habits, the findings of this study cannot be uncritically extrapolated to other institutions and settings. However, in our experience, most hospitals in Switzerland routinely conduct morning rounds in the presence of nursing staff to discuss potential overnight issues and treatment plans, thus representing an optimal timing for strategic discussions. It is also important to note that abdominal X-ray was not proposed as a ‘mandatory’ step in our algorithm, despite its predictive value for ASBO resolution. Leaving the decision to perform an X-ray to the surgeon’s discretion may represent a potential source of bias, since it was used in only four patients. The absence of a comparative pre-implementation control group may represent a further limitation. Given inherent limitations of institutional case control studies, we chose to compare our findings with the existing literature instead. Finally, we did not formally assess caregiver and patient satisfaction, which needs to be done in a next step.

In conclusion, this pilot study confirmed the protocol’s applicability and safety, highlighting the benefits of a structured approach to ASBO management from both, caregiver and patient perspective. Clinical benefits need to be further assessed in adequately powered studies.

## Electronic supplementary material

Below is the link to the electronic supplementary material.

Supplementary Material 1

## Data Availability

No datasets were generated or analysed during the current study.

## Abbreviations

ASBO: adhesive Small Bowel Obstruction
CT: Computed Tomography
NGT: nasogastric tube
ERP: enhanced recovery pathway
I-MAP: Ileus Management Protocol
LOS: Length of stay
WSC: Water soluble contrast
